# Current management strategies for patellofemoral pain: an online survey of 99 practising UK physiotherapists

**DOI:** 10.1186/s12891-017-1539-8

**Published:** 2017-05-08

**Authors:** Benjamin E. Smith, Paul Hendrick, Marcus Bateman, Fiona Moffatt, Michael Skovdal Rathleff, James Selfe, Toby O. Smith, Pip Logan

**Affiliations:** 1 Derby Teaching Hospitals NHS Foundation Trust, Physiotherapy Department (Level 3), London Road Community Hospital, Derby, DE1 2QY UK; 2 Division of Rehabilitation and Ageing, School of Medicine, University of Nottingham, Nottingham, UK; 3 Division of Physiotherapy and Rehabilitation Sciences, School of Health Sciences, University of Nottingham, Nottingham University Hospitals (City Campus), Nottingham, UK; 4 Research Unit for General Practice in Aalborg and Department of Clinical Medicine at Aalborg University, Aalborg, Denmark; 5 Department of Occupational Therapy and Physiotherapy, Department of Clinical Medicine, Aalborg University Hospital, Aalborg, Denmark; 6 Manchester Metropolitan University, Manchester, UK; 7 University of East Anglia, Norwich, UK

**Keywords:** Patellofemoral pain, Anterior knee pain, Exercise therapy, Survey

## Abstract

**Background:**

Patellofemoral pain (PFP) is considered one of the commonest forms of knee pain. This study aimed to identify how physiotherapists in the United Kingdom (UK) currently manage patellofemoral pain (PFP), particularly in relation to exercise prescription, and response to pain.

**Methods:**

An anonymous survey was designed with reference to previous surveys and recent systematic reviews. Practising UK physiotherapists who treat patients with PFP were invited to take part via an invitation email sent through professional networks, the ‘interactive Chartered Society of Physiotherapy (iCSP)’ message board, and social media (Twitter). Descriptive statistics were used to analyse the data.

**Results:**

A total of 99 surveys were completed. Responders reported a wide range of management strategies, including a broad selection of type and dose of exercise prescription. The five most common management strategies chosen were: closed chain strengthening exercises (98%); education and advice (96%); open chain strengthening exercises (76%); taping (70%) and stretches (65%). Physiotherapists with a special interest in treating PFP were statistically more likely to manage patients with orthotics (*P* = 0.02) and bracing (*P* = 0.01) compared to physiotherapists without a special interest. Approximately 55% would not prescribe an exercise if it was painful. Thirty-one percent of physiotherapists would advise patients not to continue with leisure and/or sporting activity if they experienced any pain.

**Conclusion:**

Current UK practice in the management strategies of PFP is variable. Further high quality research on which to inform physiotherapy practice is warranted for this troublesome musculoskeletal condition.

**Electronic supplementary material:**

The online version of this article (doi:10.1186/s12891-017-1539-8) contains supplementary material, which is available to authorized users.

## Background

There are over 100,000 primary care (GP) appointments a day in the UK for musculoskeletal (MSK) pain disorders [1], with associated work absenteeism costing the UK economy £7.4 billion annually [2]. Knee pain is the second most common condition, with prevalence rates estimated at between 19 and 35% in the general population [3–5]. Patellofemoral pain (PFP) is considered one of the commonest forms of knee pain [6], with an estimated prevalence of 23% in the general population [3]. It is characterised by diffuse anterior knee pain, on activities that load the joint such as squatting, running, climbing and descending stairs [6].

Long term outcomes for PFP are frequently reported as poor; a year post-diagnosis only a third of patients are pain-free [7], with 91% still reporting pain and dysfunction 4 years post-diagnosis [8]. Patients characteristically withdraw from participation in sport and leisure activities, [9]. Furthermore, individuals with PFP may develop fear, anxiety and kinesiophobia in relation to their knee pain [10–12].

Scientific consensus has not been reached in relation to aetiology [13] and there is currently a paucity of Level 1 evidence on which to base practice and treatment [14]. Various interventions have been investigated including taping, stretches, exercise, electrotherapy, joint mobilisations and foot orthoses. However systematic reviews have identified limitations in the evidence-base when drawing conclusions as to effectiveness of interventions [13, 14]. Even in relation to exercise, which has the strongest evidence-base [14], there remains insufficient evidence on which to determine the best form and dose of exercise [13].

The only previous survey of UK physiotherapy practise for PFP was in 2011 [15]; they demonstrated considerable divergence in the use of physiotherapy interventions. This survey drew participants from a small demographic area (North Wales) with a small sample of 30 participants. Therefore the generalisability of the results is limited. Subsequently, a wealth of information has been published and understanding around the concepts of chronic pain states has grown considerably [16]. More is also now understood on the impact of patients’ and therapists’ attitudes and beliefs on pain [17]. For example there is a growing body of evidence that physiotherapists with a biomedical orientation to pain are more likely to advise patients to limit their physical activity due to pain [18–20]; and consequently may induce fear-avoidant behaviours onto their patients [17, 20]. The previous survey did not include questions relating to exercise dose and pain response. In respect to these factors, and the still insufficient evidence-base on this poorly managed condition, there remains a continuing need to clearly define the range of current practice within the UK. This study therefore aimed to ascertain the current UK physiotherapy management strategies for PFP, particularly in relation to exercise prescription and therapists’ response to pain.

With reference to previous physiotherapy surveys of current practice, it is thought that physiotherapists with a special interest in PFP might have differing insights into the management strategies of this condition [21, 22]. This may lead to substantially differing approaches to exercise prescription and therapists’ response to pain during exercise and leisure activities. Therefore a secondary aim of this survey was to establish whether the level of interest in PFP influences the management strategies used.

## Methods

### Design

The study was a cross-sectional online questionnaire survey and reported following the STROBE statement [23].

### Participants

Physiotherapists were recruited via an invitation email sent through professional networks, social media (Twitter) and the ‘interactive CSP’ (iCSP) message board. The iCSP provides members with access to range of online physiotherapy communities that cover a variety of clinical and occupational interests; the survey was posted in the MSK network on the iCSP which has approximately 13,000 members. The invitation included a short summary, a link to the final survey and author contact details.

### Procedures

The survey was designed by the research team (Additional file 1), with reference to the previous PFP survey of UK practice [15]; other recent surveys of UK physiotherapy practice [21, 22]; and recent systematic reviews on conservative management strategies of PFP [14].

The survey addressed the following main areas: respondent characteristics; management strategies; exercise prescription; advice on sport and leisure activity; self-management. The survey was uploaded to Bristol Online Survey (https://nottingham.onlinesurveys.ac.uk) in July 2016, and was open until 100 respondents had completed the survey. For pragmatic reasons the number of responders was limited to 100; this reflects surveys of physiotherapy practice previously undertaken [22] and was thought to give a robust and useful amount of data. No sample size calculation was performed; our aim was large enough diversity of recruitment to ensure external validity of findings and maximum variation for specific characteristics. Full contents of the survey are included in the Additional file, ‘Additional file 1’.

### Data analysis

Data was imported into Microsoft Excel (Microsoft Corp., Redmond, WA, USA) and analysed using descriptive statistics of counts and proportions for categorical variables. Responses from physiotherapists with a special interest were compared to those without a special interest using the chi-square test, using SPSS, version 22 (IBM Corp. Armonk, NY: IBM Corp), with level of significance set at *p* < 0.05. Text responses were summarised narratively.

## Results

One hundred physiotherapists responded, with 99 completed responses from UK physiotherapists. Please see Table 1 for descriptive statistics.

**Table 1:** Descriptive survey data

Respondent Characteristics															
*n* (%)	What is your primary role? (Question 3)														
	NHS Band 5		NHS Band 6		NHS Band 7		NHS Band 8a or above		Private		Sports		Educational/Researcher		
All (*n = 99)*	8 (8.1)		28 (28.3)		15 (15.1)		9 (9.1)		27 (27.3)		6 (6.1)		6 (6.1)		
SI (*n = 33)*	2 (6.1)		10 (30.3)		6 (18.3)		2 (6.1)		9 (27.3)		2 (6.1)		2 (6.1)		
NSI (*n* = 66)	6 (9.1)		18 (27.3)		9 (13.6)		7 (10.6)		18 (27.3)		4 (6.1)		4 (6.1)		
*n* (%)	How many times do you typically see patients with PFP? (Question 11)														
	Once		Twice		3–4		5–6		7–8		9–10		10 +		
All (*n = 99)*	1 (1.0)		3 (3.0)		53 (53.5)		31 (31.3)		5 (5.1)		1 (1.0)		5 (5.1)		
SI (*n = 33)*	0 (0)		0 (0)		17 (51.5)		9 (27.3)		3 (9.1)		1 (3.0)		3 (9.1)		
NSI (*n* = 66)	1 (1.5)		3 (4.5)		36 (54.5)		22 (33.3)		2 (3.0)		0 (0)		2 (3.0)		
*n* (%)	How long would you typically expect to see patients with PFP? (Question 12)														
	Over 3 weeks		Over 6 weeks		Over 9 weeks		Over 3 months		Over 6 months		Over 12 months				
All (*n = 99)*	12 (12.1)		31 (31.3)		22 (22.2)		29 (29.3)		5 (5.1)		0 (0)				
SI (*n = 33)*	4 (12.1)		10 (30.3)		6 (18.2)		12 (36.4)		1 (3.0)		0 (0)				
NSI (*n* = 66)	8 (12.1)		21 (31.8)		16 (24.2)		17 (25.8)		4 (6.1)		0 (0)				
Management Strategies															
	What management strategies do you use for PFP? Tick all that applies? (Question 4)														
*n* (%)	Nil	Heat	Cold	Closed Chain	Open Chain	VMO	Education Advice	Stretches	Orthotics	Taping	Acupuncture	Electrotherapy	Bracing	Mobilisation	Other
All (*n = 99)*	0 (0)	5 (5.1)	13 (13.1)	97 (98.0)	75 (75.8)	56 (56.6)	95 (96.0)	64 (64.6)	53 (53.5)	69 (69.7)	11 (11.1)	5 (5.1)	9 (9.1)	54 (54.5)	10 (10.1)
SI (*n = 33)*	0 (0)	2 (6.1)	4 (12.1)	32 (97.0)	29 (87.9)*	15 (45.5)	31 (93.9)	21 (63.6)	23 (69.7)*	23 (69.7)	2 (6.1)	3 (9.1)	7 (21.2)*	18 (54.5)	6 (18.2)
NSI (*n* = 66)	0 (0)	3 (4.5)	9 (13.6)	65 (98.5)	45 (68.2)*	41 (62.1)	64 (97.0)	43 (65.2)	30 (45.5)*	46 (69.7)	9 (13.6)	2 (3.0)	2 (3.0)*	36 (54.5)	4 (6.1)
Exercise Prescription															
*n* (%)	If you prescribe exercises, how many different exercises do you prescribe at any one time? (Question 5)														
	1				2–3				4–5				6 +		
All (*n = 99)*	2 (2.0)				76 (76.8)				21 (21.2)				0 (0)		
SI (*n = 33)*	0 (0)				29 (87.9)				4 (12.1)				0 (0)		
NSI (*n* = 66)	2 (3.0)				47 (71.2)				17 (25.8)				0 (0)		
*n* (%)	If you prescribe exercises, how often do you ask them to be performed? (Question 6)														
	Every other day, or less				Once a day				Twice a day				More than twice a day		
All (*n = 99)*	15 (15.2)				34 (34.3)				34 (34.3)				16 (16.2)		
SI (*n = 33)*	7 (21.2)				11 (33.3)				11 (33.3)				4 (12.1)		
NSI (*n* = 66)	8 (12.1)				23 (34.8)				23 (34.8)				12 (18.2)		
*n* (%)	If you prescribe exercises; how many total repetitions do you usually prescribe for an exercise? (Question 7)														
	Less than 30				30–50				50 +				Patient self-directed		
All (*n = 99)*	52 (52.5)				19 (19.2)				3 (3.0)				25 (25.3)		
SI (*n = 33)*	17 (51.5)				5 (15.2)				1 (3.0)				10 (30.3)		
NSI (*n* = 66)	35 (53.0)				14 (21.2)				2 (3.0)				15 (22.7)		
*n* (%)	If you prescribe exercises, do you encourage patients to continue if they were painful? (Question 8)														
	Yes			No					Other						
All (*n = 99)*	13 (13.1)			54 (54.5)					32 (32.3)						
SI (*n = 33)*	2 (6.1)			20 (60.6)					11 (33.3)						
NSI (*n* = 66)	11 (16.7)			34 (51.5)					21 (31.8)						
Advice on Sport and Leisure Activity															
*n* (%)	Do you encourage patients to continue with their recreational/sporting activities?														
	Yes. But only if pain free.			Yes, regardless of pain.		Yes, but only with pain below a certain level			No				Other		
All (*n = 99)*	31 (31.3)			1 (1.0)		62 (62.6)			3 (3.0)				2 (2.0)		
SI (*n = 33)*	9 (27.3)			0 (0)		23 (69.7)			1 (3.0)				0 (0)		
NSI (*n* = 66)	22 (33.3)			1 (1.5)		39 (59.1)			2 (3.0)				2 (3.0)		
Self-Management															
*n* (%)	Do you expect patients to: (Question 10)														
	Self-Manage after 1st Appt’			Self-Manage with guidance					Self-Manage with physio led treatment				Attend for regular physio led treatment		
All (*n = 99)*	2 (2.0)			40 (40.4)					50 (50.5)				7 (7.1)		
SI (*n = 33)*	0 (0)			12 (36.4)					20 (60.6)				1 (3.0)		
NSI (*n* = 66)	2 (3.0)			28 (42.4)					30 (45.5)				6 (9.1)		

All values are reported as actual number of responders. Values in parenthesis indicate percentages

*All* all responders to the survey, *SI* physiotherapists with a special interest, *NSI* physiotherapists without a special interest, *NHS* National Health Service

*Statistically significant difference between physiotherapists with a special interest and physiotherapists without a special interest (*p* < 0.05)

### Respondent characteristics

**Fig. 1:**
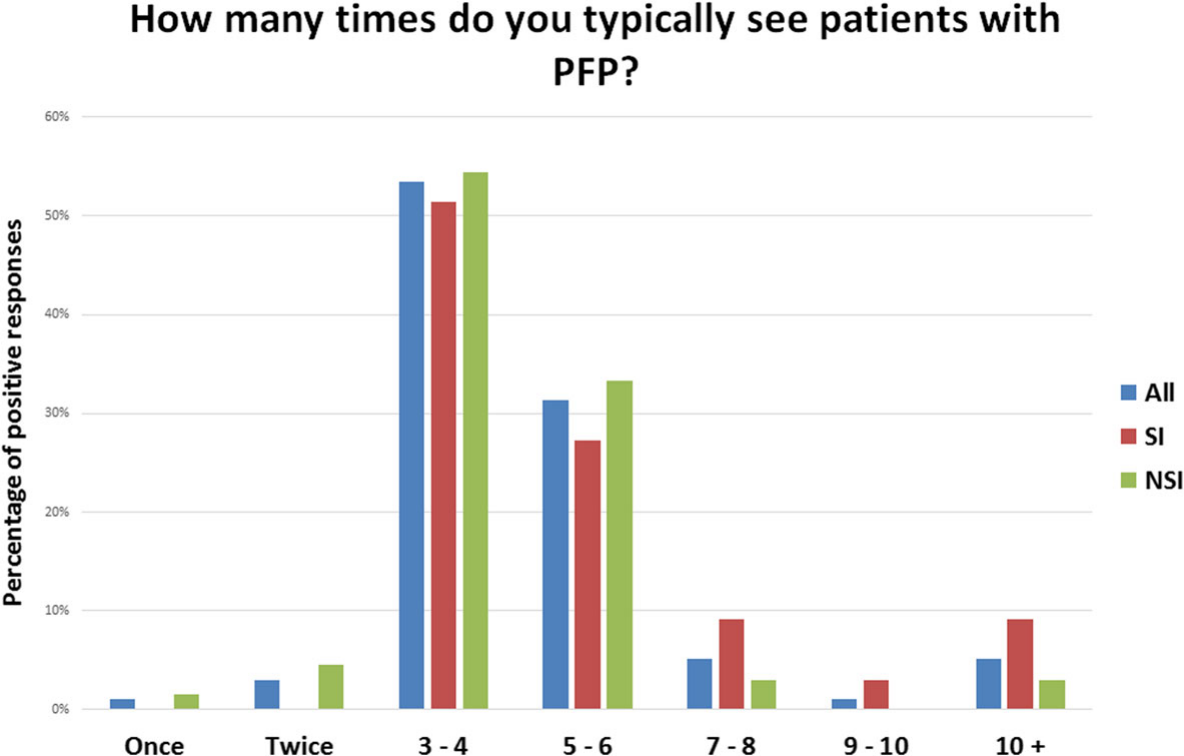
Number of appointments. All, all responders to the survey; SI, physiotherapists with a special interest; NSI, physiotherapists without a special interest

**Fig. 2:**
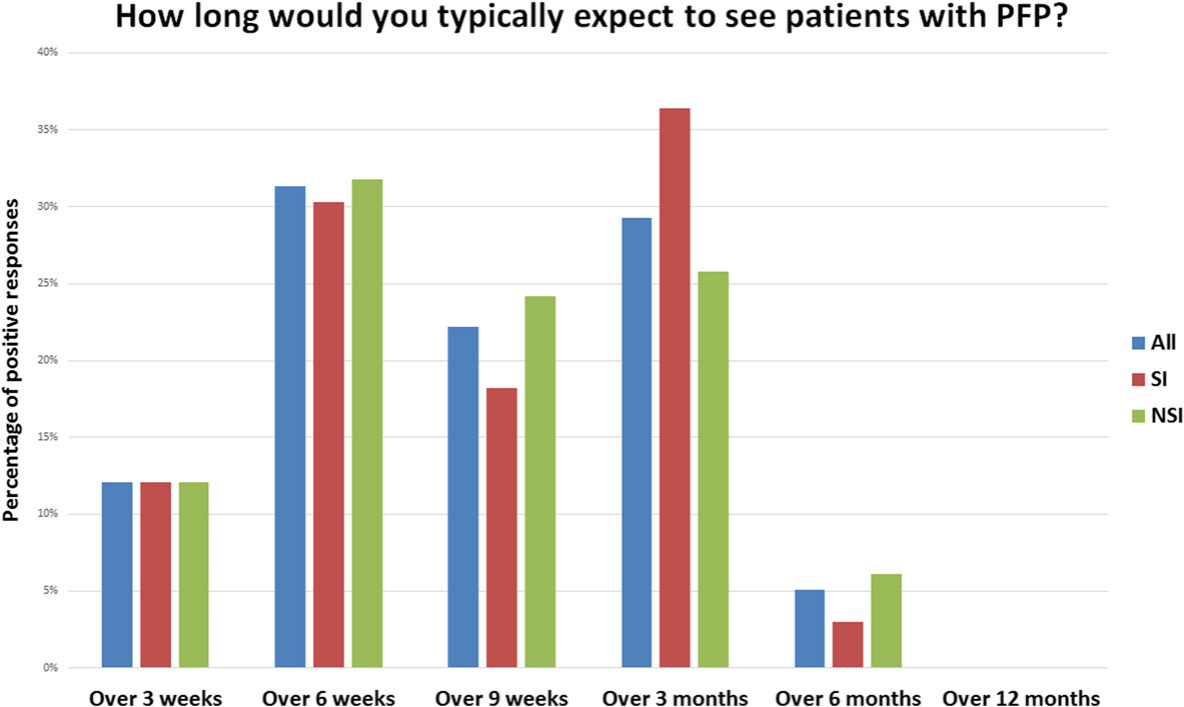
How long would you typically expect to see patients with PFP? All, all responders to the survey; SI, physiotherapists with a special interest; NSI, physiotherapists without a special interest

- *Do you have a special interest in treating patellofemoral pain? (Question 2)*
  Thirty three (33.3%) physiotherapists responded they had a special interest in PFP, and 66 (66.7%) responded they did not.
- *What is your primary role? (Question 3)*
  Within the NHS most UK physiotherapists are employed on national Agenda for Change contracts, with band 5 level broadly representing junior qualified physiotherapists. Generally, the level of banding increases in line with the level of seniority, up to band 8. In general there was a wide variety of roles, across different levels and settings. The most common three settings were NHS Band 6 (28%), Private Practice (27%) and NHS Band 7 (15%). There was no significant difference in primary role between those with a special interest and those without (χ2 = 1.121 *p* = 0.98).
- *How many times do you typically see patients with PFP? (Question 11)*
  There was a great variation in the number of treatment sessions provided by the UK physiotherapists, ranging from 1 to 10+ appointments (Fig. 1). There was no statistically significant difference between physiotherapists with and without a special interest (χ2 = 7.496 *p* = 0.28).
- *How long would you typically expect to see patients with PFP? (Question 12)*
  The vast majority of UK physiotherapists (95%) within this study would expect to see patients for no more than 6 months (Fig. 2). There was no statistically significant difference between physiotherapists with and without a special interest (χ2 = 1.624 *p* = 0.80).

### Management strategies

**Fig. 3:**
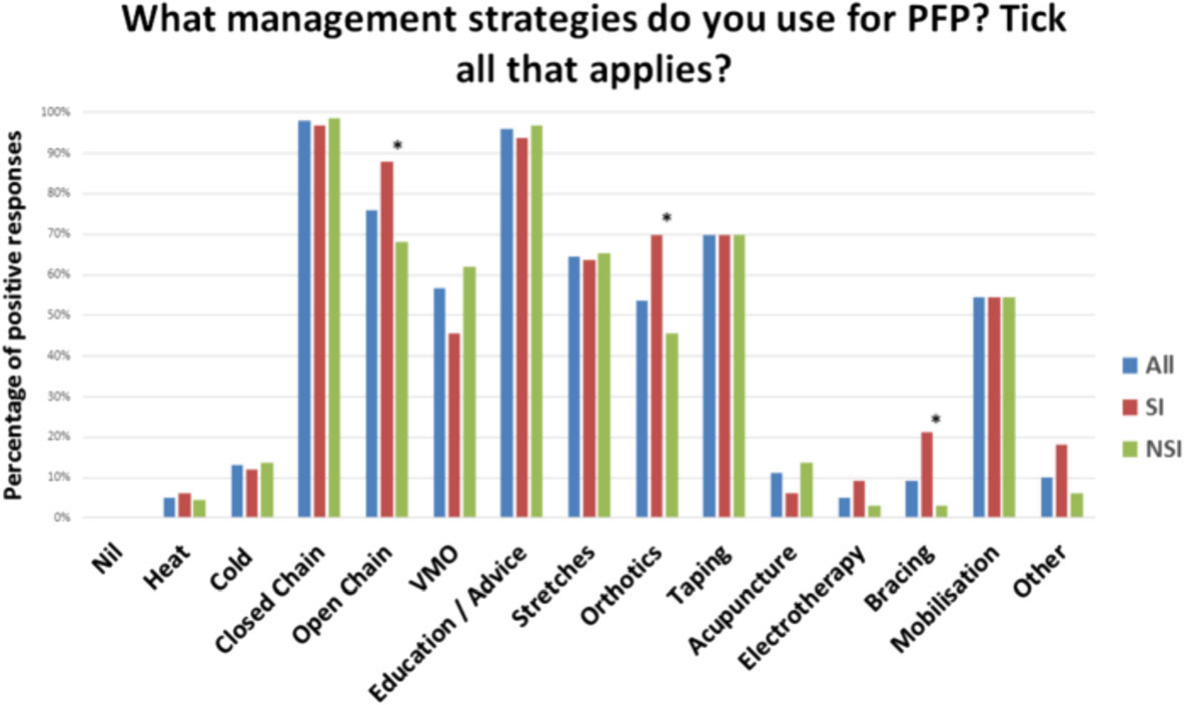
What management strategies do you use for PFP? All, all responders to the survey; SI, physiotherapists with a special interest; NSI, physiotherapists without a special interest; *, statistically significant difference between physiotherapists with a special interest and physiotherapists without a special interest (*p* < 0.05)

- *What management strategies do you use for PFP? Tick all that applies? (Question 4)*
  UK physiotherapists currently offer their patients a wide variety of treatment options (Fig. 3). The five most common options chosen were: close chain strengthening exercises (98%); education and advice (96%); open chain strengthening exercises (76%); taping (70%) and stretches (65%). Responders with a declared special interest in PFP were more likely to prescribe open chain exercises (88%, 95% CI 73–95% versus 69%, 95% CI 56–78% ); orthotics (70%, 95% CI 53–83% versus 46%, 95% CI 34–57%) and bracing (21%, 95% CI 11–38% versus 3%, 95% CI 1–10%); these differences were statistically significant (χ2 = 3.960 *p* = 0.04; χ2 = 5.198 *p* = 0.02; χ2 = 8.800 *p* = 0.01 respectively). The pattern of responses was closely matched between those with a special interest and those without for the remainder of management options (*p* > 0.05). Ten responders specified ‘other’. Responses included: deep transverse friction, soft tissue massage, foam rolling and myofascial release.

### Exercise prescription

**Fig. 4:**
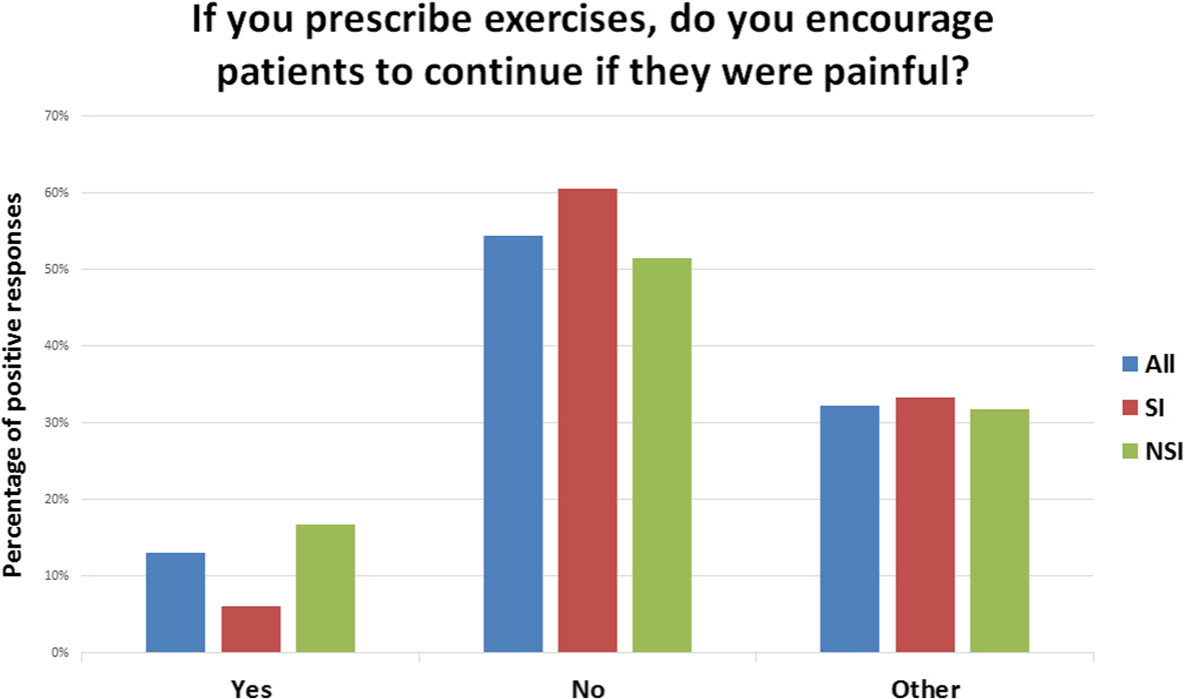
If you prescribe exercises, do you encourage patients to continue if they were painful? All, all responders to the survey; SI, physiotherapists with a special interest; NSI, physiotherapists without a special interest

- *If you prescribe exercises, how many different exercises do you prescribe at any one time? (Question 5)*
- *If you prescribe exercises, how often do you ask them to be performed? (Question 6)*
- *If you prescribe exercises; how many total repetitions do you usually prescribe for an exercise? (Question 7)*
  There was a wide variety of total number of exercises prescribed, with physiotherapists offering between 1 and 5 exercises; with differing number of total repetitions. There was also a wide variety in how often the exercises were to be completed, from every other day up to more than twice a day. There was no significant difference between those with a special interest and those without (χ2 = 3.725 *p = 0.16;* χ2 = 1.729 *p = 0.63;* χ2 = 7.496 *p = 0.28 respectively*).
- *If you prescribe exercises, do you encourage patients to continue if they were painful? (Question 8)*
  A greater number of physiotherapists with a declared special interest in PFP responders reported that they would not encourage patients to continue if the exercises were painful (61%, 95% CI 44–75% versus 52%, 95% CI 40–63%) (Fig. 4). However, this difference was not statistically significant (χ2 = 2.234 *p* = 0.33). Thirty two physiotherapists indicated ‘other’ and all 32 qualified their answers by completing the comment box. The criteria for continuing with the exercise differed among therapists, with some suggesting they would continue if the exercises were: less than a certain level of pain measured, with answers ranging from 2/10 to 4/10; only moderately painful; acceptable to the patient; dependent on severity and irritability or that it would vary from patient to patient. No pain scale was offered, but all comments used the visual analogue scale 0–10. Responders who answered ‘yes’ or ‘no’ weren’t given the option of leaving a comment.

### Advice on sport and leisure activity

**Fig. 5:**
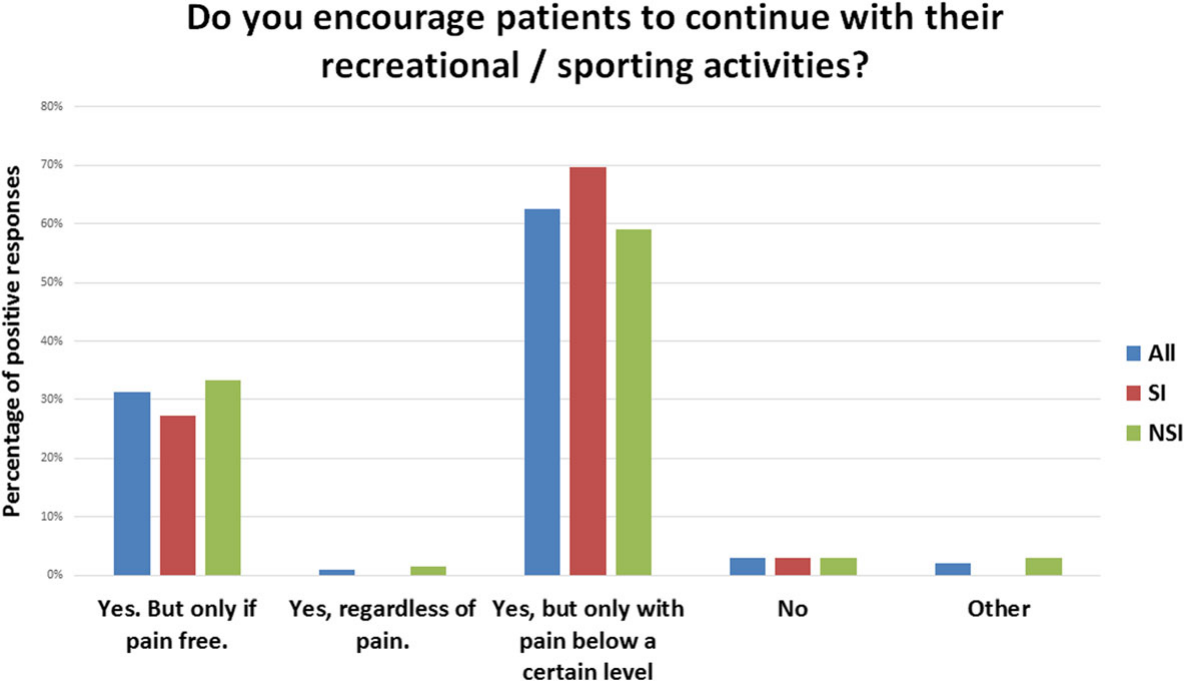
Do you encourage patients to continue with their recreational/sporting activities? All, all responders to the survey; SI, physiotherapists with a special interest; NSI, physiotherapists without a special interest

- *Do you encourage patients to continue with their recreational/sporting activities? (Question 9)*
  The majority of UK physiotherapists (93.7%; 97% with a special interest; 93% without a special interest) in this study would only encourage patients to continue with leisure and sporting activity if it was pain free, or if the pain was below a certain level (Fig. 5). There was no significant difference between those with a special interest and those without (χ2 = 2.153 *p* = 0.71). Forty-seven respondents qualified their answers in a variety of ways, suggesting they would encourage the patient to continue if: the pain was less than a certain level of pain measured on the visual analogue scale, with answers ranging from 2/10 to 6/10; whether the pain settled immediately; the pain settled within a few hours; the pain settled the same day; the pain settled within 24 h; dependent on severity and irritability or that it would vary from patient to patient. No pain scale was offered, but all comments that used one used the visual analogue scale 0–10.

### Self-management

**Fig. 6:**
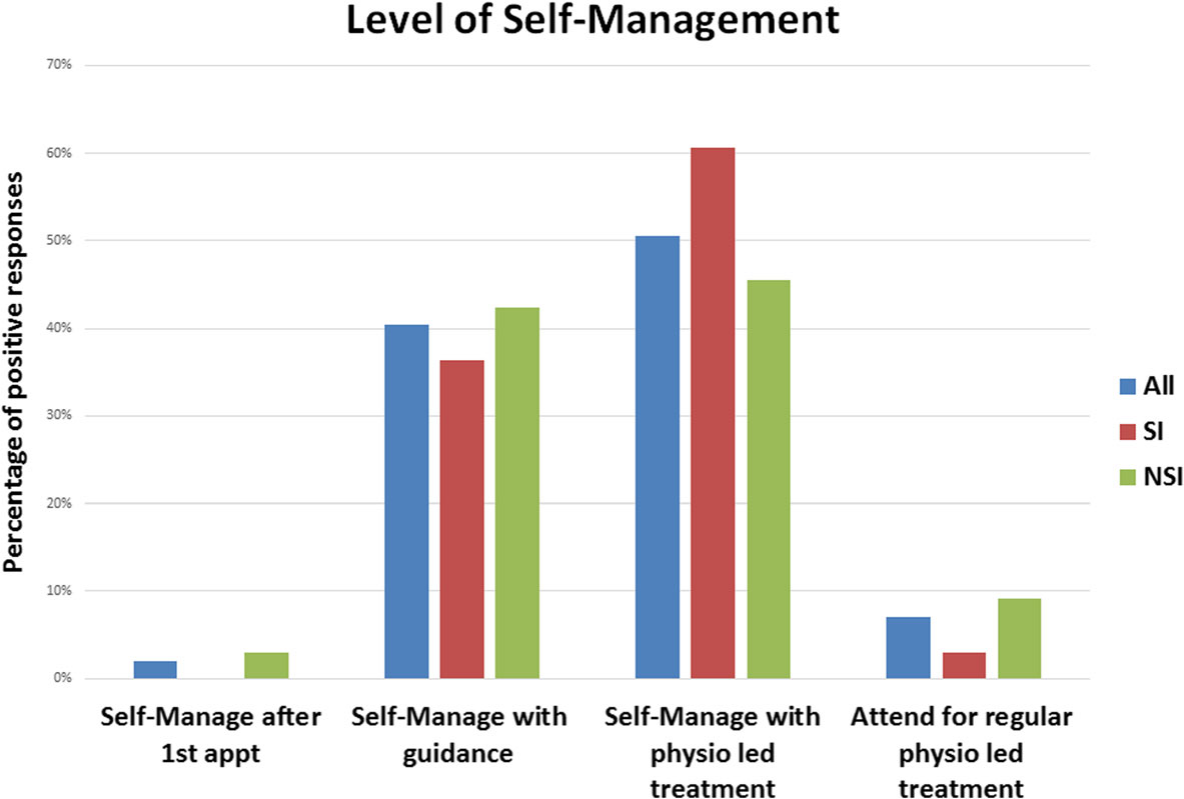
Level of self-management. All, all responders to the survey; SI, physiotherapists with a special interest; NSI, physiotherapists without a special interest

- *Do you expect patients to self-manage? (Question 10)*
  There was no significant difference between physiotherapists with and without a special interest (χ2 = 3.347 *p* = 0.34) (Fig. 6). A greater number of physiotherapists would expect patients to self-manage with physiotherapist led treatments, compared with physiotherapists who would expect self-management with physiotherapy guidance (51%, 95% CI 41–60% versus 40%, 95% CI 31–50%).

### Any additional comments

- *Any additional comments? (Question 13)*
  All responders were given an open text box at the end of the survey to leave any additional information they felt necessary, with 24 leaving a comment. The majority of comments gave further information in relation to management approaches, and identified that most physiotherapists vary their treatment method depending on the patient; suggesting an individualised approach based on age, severity of symptoms, duration of symptoms, patient’s beliefs and previous treatments. Three other responses had further information on work setting, comprising: working with children; professional athletes; and amateur athletes.

## Discussion

This paper describes a sample of UK physiotherapists who treat PFP in terms of their level of self-declared interest and setting. It identifies and quantifies the management strategies used, exercise prescription parameters and perceived likely treatment length. A total of 99 responses were gained from a broad sphere of UK physiotherapists, with variable experience and practice settings. There was no difference in the proportion of physiotherapists with or without a special interest in PFP across the difference practice settings. The physiotherapists in this sample currently offer a wide variety of interventions; and provide a wide variety of education and advice in response to pain. The amount of variability in how physiotherapists treat PFP might in part reflect the lack of sufficient clinical guidelines; and/or the uncertainty and lack of sufficient Level 1 evidence on which to base practice.

In terms of management strategies and treatment options the results indicate that advice/education and exercise seem to be the mainstay of treatment, although the actual prescription parameters vary considerably; there was no consistency with regards to the number of different exercises prescribed, total number of repetitions and how frequently they should be performed. Possible dissonance between research and practice is demonstrated with reference to the passive interventions: taping, orthotics, bracing, and mobilisation, with a recent systematic review of systematic reviews highlighting no Level 1 evidence to support their use in the long term [14]. However respondents were not given the opportunity to specify if treatments were employed short-term or long-term. An observation from the results is that physiotherapists with a special interest in treating PFP are statistically more likely to manage patients with orthotics (70%, 95% CI 53–83% versus 46%, 95% CI 34–57%) and bracing (21%, 95% CI 11–38% versus 3%, 95% CI 1–10%) than physiotherapists without a special interest. The reason for this difference is unclear.

Approximately 55% of physiotherapists within this sample (who would prescribe an exercise) would not prescribe an exercise if it was painful. Though not statistically significant, this proportion is higher in physiotherapists with a special interest in PFP (61%, 95% CI 44–75% versus 52%, 95% CI 40–63%). Following a similar theme, 31% of the physiotherapists would advise patients not to continue with leisure and/or sporting activity if the patient experienced any pain. Many of the physiotherapists within this sample qualified their answers by stating an upper level of pain they would remain comfortable with whilst encouraging the patient to continue with their exercise, leisure and/or sporting activity, with a wide range of pain scores and phrases employed. This belief may predominate from historical clinical reasoning labelling one major cause of PFP as patella mal-tracking/malalignment [24]. Current thinking in relation to understanding chronic and persistent pain states, and the importance of patients’ and therapists’ attitudes and beliefs, challenges the view that patients should avoid painful activity [16, 17, 25, 26]. This is an important element when considering: 23% of patients with PFP will stop participating in physical activity because of their knee pain [9]. Physical inactivity accounts for one in six deaths in the UK [27] and costs an estimated £7.4 billion a year in England through direct costs of treating lifestyle related disease and indirect costs of sickness absence [28]. Furthermore, there have been some positive results with painful loaded exercises for tendon pain [29], shoulder pain [30–32], low back pain [33] and plantar heel pain [34]. A recent systematic review and meta-analysis of painful exercises versus pain free exercises for chronic MSK pain found protocols using painful exercises offered a small, but significant benefit over pain-free exercises in the short term; at long term there was no difference [35]. The results of the review indicated that pain need not be a barrier to successful outcomes, and protocols using painful exercises typically have higher loads and dose of exercise. These results have been supported in a PFP study in Norway (*n* = 42) investigating different dosages of exercise intervention; a high dose regime versus a low dose regime [36]. The results demonstrated significant benefit of the high dose in terms of pain and function at 12 weeks. This difference was even greater at 1 year follow-up, as the high dose group continued to improve in terms of pain and function, while the low dose group had relapsed [36, 37]. Pain and dose response as an aspect of exercise prescription clearly warrants further investigation.

The range of responses provided by the physiotherapists within this survey in relation to the number of predicted appointments and length of treatment was diverse. Comparing these results to prognostic data within the literature, a systematic review and meta-analysis indicate that exercise interventions will improve pain at long term (>12 weeks) by 2.1 to 19.3 mm on a zero to 100 mm VAS. Strikingly, pooled data indicates that exercise interventions over no treatment will result in only 88 more patients (95% CI two fewer to 210 more) per 1000 reporting a clinically important improvement in their pain in the long term (>12 weeks) [13]. Contrast this with the two largest randomised controlled trials on adults, Van Linschoten et al. [38] and Collins et al. [39], it can be seen that between 51 and 81% of patients reported successful outcomes at 12 months follow-up (defined as ‘completely recovered or strongly improved’ and ‘moderate or marked improvement’ on a seven and five-point Likert scale respectively). The large variability in answers provided to our survey seems to reflect the level of uncertainty of prognosis prediction within the literature [13, 38, 39].

### Strengths and limitations

Our main aim was to understand the management strategies used by UK physiotherapists for PFP. Other methods for gaining this knowledge could have included the use of focus groups, notes audits, paper surveys, or online survey with the addition of a case study vignette [22]. It is possible that the use of a vignette may have resulted in a different data set, as it is thought that they may clarify and explore attitudes and beliefs more fully [40]. Additionally, it would have been interesting to note if physiotherapists’ geographical location and length of time qualified had an impact on responses; or if physiotherapists prescribed a certain intensity of exercise, however these data weren’t collected.

Recruitment for this survey was limited to 6 months, however for pragmatic reasons the number of responders was limited to 100; this is consistent with previous surveys of physiotherapy practice [22], however is a major limitation of the study. A larger sample size better representing the UK physiotherapy population may improve the generalisability of the results. It is worth noting though, that a wide range of practice settings, including different levels of NHS banding, were represented in the sample of 99 UK physiotherapists, and therefore how much more information would have been obtained from a larger size is questionable.

A strength of this study is that despite the survey being open for 6 months, it reached its target of 100 responders within 1 day. It is unknown the number of physiotherapists recruited from each electronic mail shot; iCSP, twitter or email. Social media is a relatively new form of communication for professional networking and professional development, and it may be that the form of communication this survey used unfavourably biased recruitment towards typical ‘early adopters’ of technology, biasing the results in favour of ‘early adopters’ of health research. However, it is worth noting the wide range of management strategies and exercise prescription used by the sample of physiotherapists within this survey. It is unknown the number of physiotherapists who received invitation emails, tweets or messages on the iCSP, and therefore the total number of physiotherapists sampled is unknown.

For unknown reasons one respondent indicated they were not a UK physiotherapist, but nonetheless went on to complete the survey. This participant’s answers were removed from the dataset, leaving 99 completed responses from UK physiotherapists.

## Conclusion

There appears no standardised method for treatment and management of PFP in the UK in relation to exercise prescription and therapists’ response to pain during exercise and leisure activities. Responders in this survey stated they would undertake: a wide range of management strategies, including exercises prescriptions and dosage; differing degrees of education and advice with regards to continuing with leisure and sporting activity; and offer a broad prediction in physiotherapy appointment frequency and duration. This indicates that current UK practice in the management of PFP is widely variable. This variability might reflect the individualised treatment approach traditional physiotherapy assessments and treatments use [24, 41]; or could also reflect the level of uncertainty and lack of sufficient level 1 evidence on which to base practice. Further high quality research on exercise prescription in relation to pain mechanisms and dose response is clearly warranted for this persistent and troublesome problem. In addition, detailed qualitative work exploring the rationale behind physiotherapists’ beliefs and attitude to pain and exercise prescription will help advance future research into exercise interventions.

## Additional file

Additional file 1: Survey Content. (DOCX 14 kb)

## Abbreviations

CI: Confidence Interval
GP: General Practitioner
iCSP: interactive Chartered Society of Physiotherapy
MSK: Musculoskeletal
NHS: National Health Service
NSI: Physiotherapists without a special interest
PFP: Patellofemoral pain
SI: Physiotherapists with a special interest
UK: United Kingdom
VMO: Vastus medialis oblique
