# Supplementary material for: Current management strategies for patellofemoral pain: an online survey of 99 practising UK physiotherapists
Source: BMC Musculoskelet Disord. 2017 May 8;18:181. doi: 10.1186/s12891-017-1539-8 (PMC5422884; doi:10.1186/s12891-017-1539-8)
Supplement: Additional file 1: — Survey Content. (DOCX 14 kb) [file 12891_2017_1539_MOESM1_ESM.docx]

**Additional File 1 - Survey Content**

1. Are you a UK based physiotherapist that regularly sees patients with patellofemoral pain?
   1. Yes – please continue
   2. No – thank you for your time, please exit the survey
2. Do you have a specialist interest in treating patellofemoral pain?
   1. Yes
   2. No
3. What is your primary role?
   1. NHS Band 5
   2. NHS Band 6
   3. NHS Band 7
   4. NHS Band 8a or above
   5. Private Practice
   6. Sport Club / Elite Athletes
   7. Educational / Research
4. What management strategies do you use for PFP? Tick all that applies?
   1. No treatment needed
   2. Heat treatment
   3. Cold treatment
   4. Muscle strengthening - closed chain
   5. Muscle strengthening - open chain
   6. VMO exercises
   7. Education / Advice
   8. Stretching
   9. Foot orthotics
   10. Taping
   11. Acupuncture
   12. Electrotherapy
   13. Bracing
   14. Mobilisations
   15. Other (please specify)
5. If you prescribe exercises, how many different exercises to you prescribe at any one time?
   1. 1
   2. 2-3
   3. 4-5
   4. 6+
6. If you prescribe exercises, how often do you ask them to be performed?
   1. Every other day, or less
   2. Once a day
   3. Twice a day
   4. More than twice a day
7. If you prescribe exercises; how many total repetitions do you usually prescribe for an exercise?
   1. Less than 30
   2. 30 – 50
   3. 50+
   4. Patient self-directed
8. If you prescribe exercises, do you encourage patients to continue if they were painful? Please qualify your answer
   1. Yes
   2. No
   3. Other

Further comments:

1. Do you encourage patients to continue with their recreational / sporting activities
   1. Yes. But only if pain free.
   2. Yes, regardless of pain.
   3. Yes, but only with pain below a certain level (please qualify)
   4. No
   5. Other

Further comments:

1. Do you expect patients to (tick all that applies):
   1. Self-Manage after the first appointment
   2. Self-Manage with follow-up appointments for guidance
   3. Self-Manage with follow-up appointments for physiotherapy led treatment
   4. Not self-manage, but attend regular physiotherapy led treatment sessions
2. How many times do you typically see patients with PFP?
   1. Once
   2. Twice
   3. 3 – 4 times
   4. 5 – 6 times
   5. 7 – 8 times
   6. 9 – 10 times
   7. More than 10 times
3. How long would you typically expect to see patients with PFP?
   1. Over 3 weeks
   2. Over 6 weeks
   3. Over 9 weeks
   4. Over 3 months
   5. Over 6 months
   6. Over 12 months
4. Any additional comments:
